# Hepatic Duct Adenoma Identified Using Direct Cholangioscopy

**DOI:** 10.7759/cureus.57424

**Published:** 2024-04-01

**Authors:** Butros Fakhoury, Iyiad Alabdulrazzak, Michael Talanian, Syed Mahmood

**Affiliations:** 1 Internal Medicine, St. Elizabeth's Medical Center, Boston, USA; 2 Gastroenterology, Tufts Medical Center, Boston, USA; 3 Gastroenterology, St. Elizabeth's Medical Center, Boston, USA

**Keywords:** high-grade dysplasia, common hepatic duct, choledocholithiasis, direct cholangioscopy, bile duct adenomas

## Abstract

Bile duct adenomas (BDAs) are rare benign tumors that can arise in the intra-hepatic or extra-hepatic biliary tree. We present a case of a 46-year-old female who presented with symptoms suggestive of choledocholithiasis. Direct cholangioscopy identified a 15 mm polypoid lesion in the common hepatic duct (CHD). Biopsy revealed a BDA. We present this case to highlight the role of direct cholangioscopy in the diagnosis and management of BDAs.

## Introduction

Bile duct adenomas (BDAs) are rare benign tumors that can arise within the intra-hepatic or extra-hepatic biliary system. Clinical manifestations can vary, ranging from asymptomatic incidental findings and biliary cholic with obstructive jaundice, to alarming symptoms that can mimic malignancy [1]. The diagnosis of BDAs poses a challenge and is often mistaken for choledocholithiasis or cholangiocarcinoma when identified using conventional imaging techniques. We present a case of a 46-year-old female who had symptoms suggestive of choledocholithiasis. Direct cholangioscopy revealed a 15 mm polypoid lesion in the common hepatic duct (CHD) and biopsy confirmed a BDA.

## Case presentation

**Figure 1:**
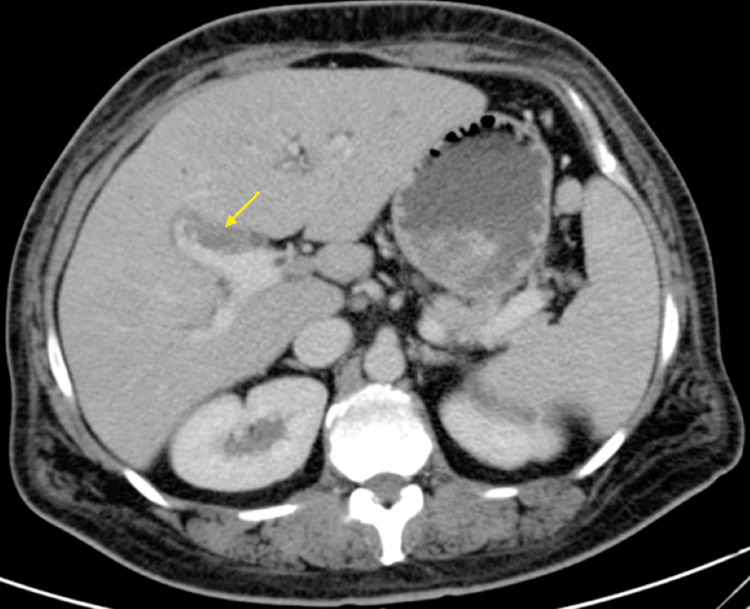
CT abdomen and pelvis with intravenous contrast showing intra-hepatic biliary dilatation and filling defect at the level of the CHD extending to the right hepatic duct. CT: computed tomography, CHD: common hepatic duct

**Figure 2:**
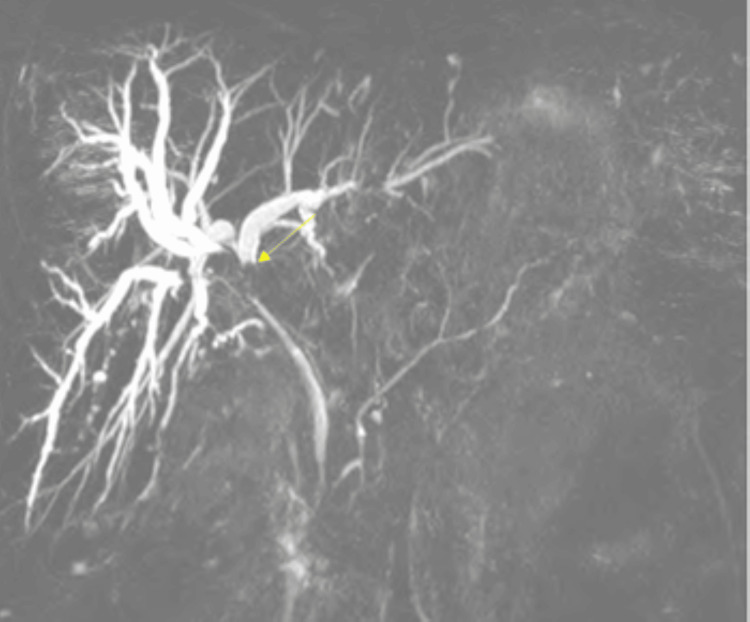
MRCP illustrating dilated intra-hepatic bile ducts with a filling defect at the hepatic hilum. MRCP: magnetic retrograde cholangiopancreatography

**Figure 3:**
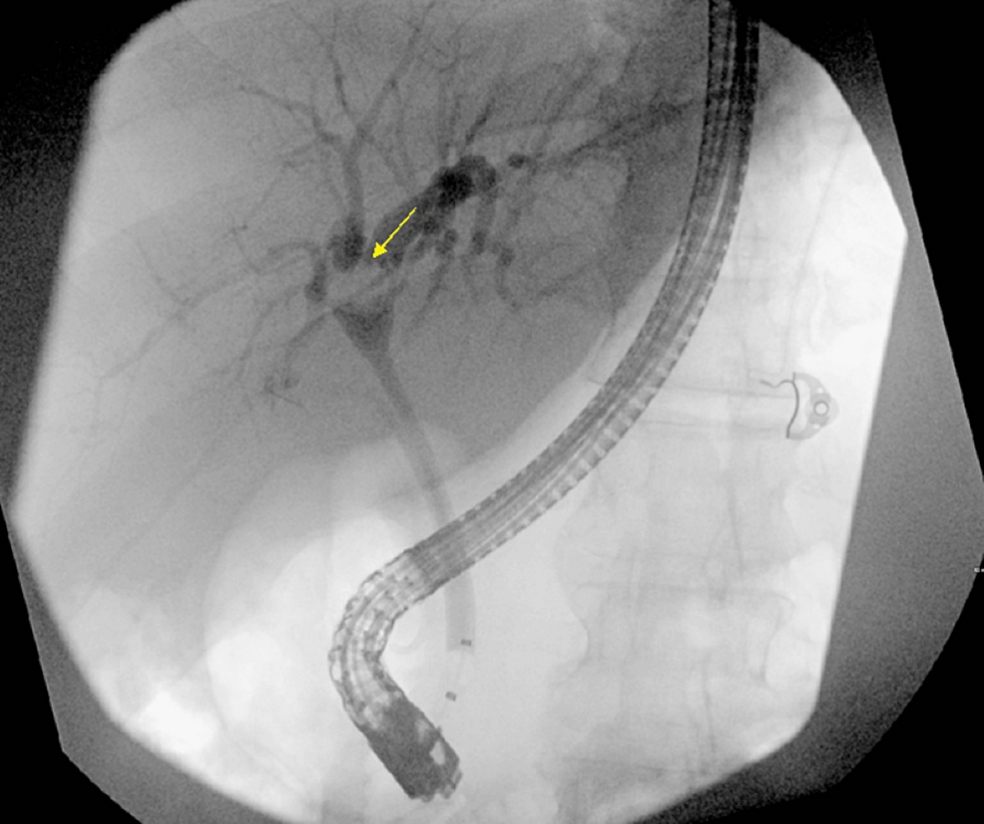
ERCP demonstrating a filling defect at the level of the CHD. ERCP: endoscopic retrograde cholangiopancreatography, CHD: common hepatic duct

**Figure 4:**
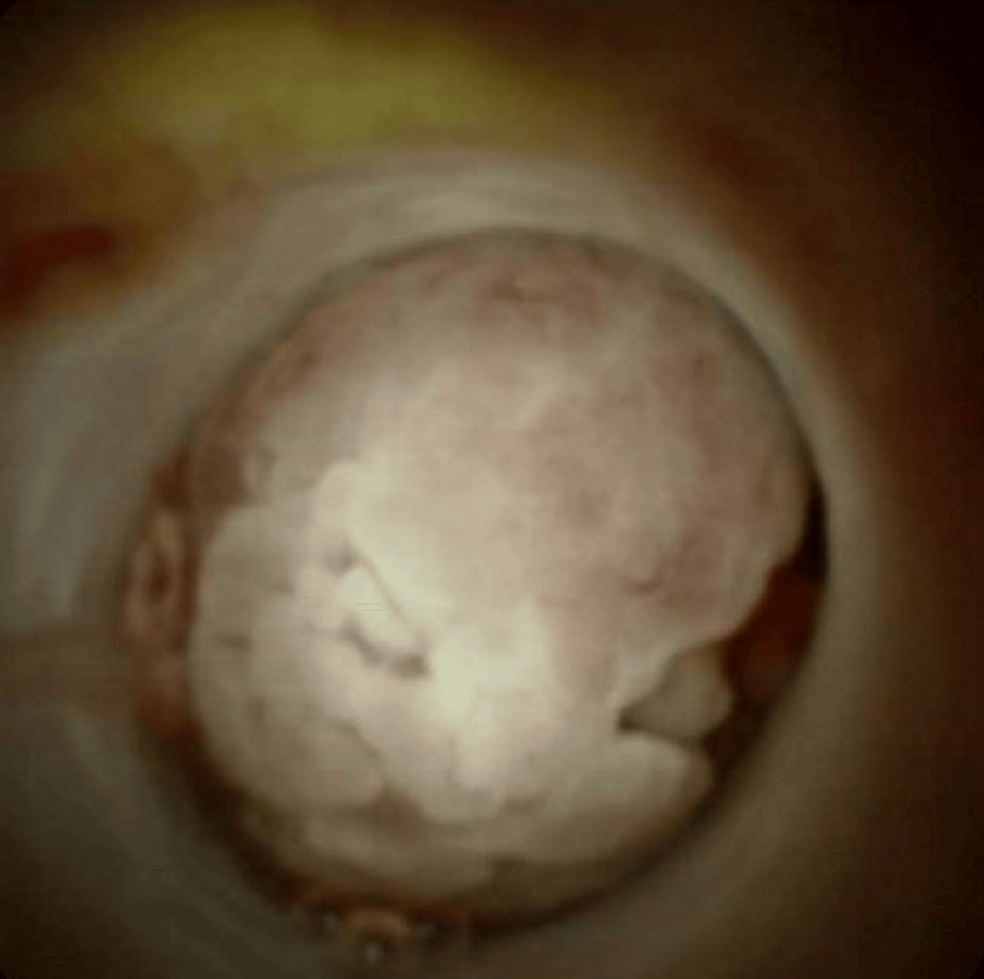
Polypoid mass measuring 15 mm noted in the common hepatic duct, 1 cm distal to the bifurcation using SpyGlass cholangioscopy.

**Figure 5:**
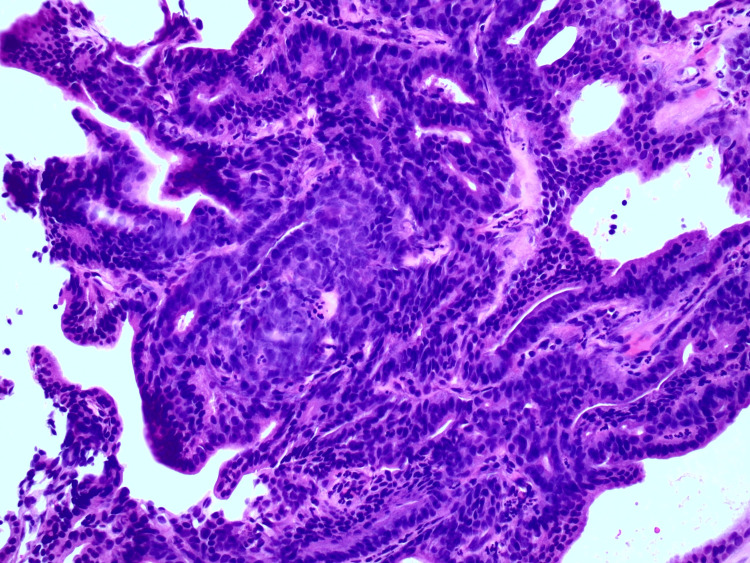
Forceps biopsy depicting hyperchromatic adenomatous epithelium with preserved architecture consistent with cholangioadenoma.

A 46-year-old female with a medical history notable for cholecystectomy presented at the hospital complaining of intermittent epigastric abdominal pain persisting for the past two months, associated with nausea, vomiting, and diffused pruritis. Physical examination was unremarkable. Liver function tests revealed a cholestatic pattern of liver injury. A computed tomography (CT) scan of the abdomen revealed intrahepatic biliary dilatation with a polypoid filling defect at the level of the CHD. This finding raised concerns about either a solid mass or choledocholithiasis (Figure 1). Magnetic retrograde cholangiopancreatography (MRCP) revealed intrahepatic biliary dilatation with transition zone at the level of the right and left ductal junction, as well as soft tissue density within the proximal CHD extending into the right biliary duct. Based on these findings, the differential diagnosis leaned toward a soft tissue mass, with considerations for either cholangiocarcinoma or cholangioadenoma, rather than choledocholithiasis (Figure 2). Subsequent endoscopic retrograde cholangiopancreatography (ERCP) demonstrated a large well-defined circular filling defect at the bifurcation of the right and left hepatic ducts. The biliary tree was swept with a 12 mm balloon resulting in minimal sludge (Figure 3). Due to the high suspicion of a soft tissue mass, SpyGlass cholangioscopy was performed demonstrating a 15 mm polypoid mass in the CHD, located at 1 cm distal to the biliary bifurcation (Figure 4). Forceps biopsies revealed BDAs (Figure 5). The patient was then referred to hepatobiliary surgery, where she underwent a successful surgical excision.

## Discussion

Extra-hepatic biliary tumors can be categorized into benign, premalignant, and malignant lesions. Among these, benign epithelial lesions of the bile duct constitute approximately 6% of the extra-hepatic biliary lesions. These benign epithelial lesions can be further classified into various subtypes, including BDAs, biliary adenofibroma, and mucinous cystic neoplasms of the liver and biliary system [2]. Extra-hepatic BDAs are commonly found in the common bile duct (CBD) (64%), followed by CHD (18%), and the cystic duct (8%) [3].

The scarcity of BDAs presents diagnostic challenges, potentially resulting in misinterpretation of conventional imaging studies and misdiagnosed as stones, sludge, or cholangiocarcinoma [1]. Xu et al. proposed that endoscopic ultrasound can be more effective in identifying benign biliary lesions, as they typically exhibit hypervascularity and homogeneous echogenicity [4]. Definitive diagnosis requires a biopsy, often obtained intra-operatively. Recently, direct cholangioscopy has emerged as a valuable tool offering both diagnostic and therapeutic benefits that can guide surgical management [5]. Eccles et al. reported a case series demonstrating the utility of cholangioscopy, where synchronous CBD cholangiocarcinoma and hilar cholangioadenoma were identified, altering surgical management based on findings not visualized in prior imaging studies [6]. A literature review by Loh et al. identified 39 cases of BDAs, with the majority diagnosed via surgical specimens, and only a limited number of cases diagnosed based on biopsies obtained through direct cholangioscopy [7]. 

## Conclusions

Our case highlights the significance of direct cholangioscopy in diagnosing and managing BDAs. Furthermore, the therapeutic role of direct cholangioscopy has been explored in a limited number of case reports. Consequently, additional research is required to better understand the role of endoscopic excision of benign lesions, particularly in the proximal extra-hepatic bile duct system where accessibility is limited. 
